# Containing Chaos: A Self-Reflection of a Final Year Medical Student’s Elective in a High Security Psychiatric Hospital

**DOI:** 10.1192/j.eurpsy.2022.1540

**Published:** 2022-09-01

**Authors:** R. Nieuwoudt

**Affiliations:** Sheffield Health and Social Care, Home Treatment Team, Sheffield, United Kingdom

**Keywords:** undergraduate, reflection, Elective, forensic

## Abstract

**Introduction:**

Throughout training, medical students are often only exposed to a limited selection of psychiatric specialities, predominantly general adult inpatient settings. This medical student had the opportunity to undergo a placement at a high security forensic hospital. With only three such hospitals in England, this is an environment that few students and even qualified doctors have been able to experience. In this presentation, the author will explore their prior expectations, key skills gained, and surprising realisations that made the elective highly valuable.

**Objectives:**

To reflect on the skills learned and revelations made during the elective period and share these as a presentation.

**Methods:**

The author completed a 6-week placement at Ashworth High Security Psychiatric Hospital. He then reflected on his experiences.

**Results:**

This placement allowed the development of a range of skills and personal discoveries. The skills included enhanced personal safety awareness, improved use of varied communication styles, and de-escalation and management techniques with higher risk patients. The main finding was the fine line between Ashworth’s patients and mainstream society, and how easily these two entities can overlap. *Carl Jung* spoke of a ‘shadow’ that must be integrated, and the humanity within each patient made this philosophical concept a sobering reality.

**Conclusions:**

High security placements are valuable educational opportunities and teach important skills, not often found in the current medical school curriculum. These placements offer the transferable communication and interpersonal skills essential in any budding psychiatrist, and also provide a vital environment for self-reflection and personal growth.

**Disclosure:**

No significant relationships.

